# Investigation of bidirectional longitudinal associations between advanced epigenetic age and peripheral biomarkers of inflammation and metabolic syndrome

**DOI:** 10.18632/aging.101992

**Published:** 2019-06-07

**Authors:** Filomene G. Morrison, Mark W. Logue, Rachel Guetta, Hannah Maniates, Annjanette Stone, Steven A. Schichman, Regina E. McGlinchey, William P. Milberg, Mark W. Miller, Erika J. Wolf

**Affiliations:** 1 National Center for PTSD at VA Boston Healthcare System, Boston, MA 02130, USA; 2 Department of Psychiatry, Boston University School of Medicine, Boston, MA 02118, USA; 3 Biomedical Genetics, Boston University School of Medicine, Boston, MA 02118, USA; 4 Pharmacogenomics Analysis Laboratory, Research Service, Central Arkansas Veterans Healthcare System, Little Rock, AR 72205, USA; 5 Geriatric Research Educational and Clinical Center and Translational Research Center for TBI and Stress Disorders, VA Boston Healthcare System, Boston, MA 02130, USA; 6 Department of Psychiatry, Harvard Medical School, Boston, MA 02115, USA

**Keywords:** epigenetic age, DNA methylation age, longitudinal, C-reactive protein, metabolic syndrome

## Abstract

Epigenetic age estimations based on DNA methylation (DNAm) can predict human chronological age with a high level of accuracy. These DNAm age algorithms can also be used to index advanced cellular age, when estimated DNAm age exceeds chronological age. Advanced DNAm age has been associated with several diseases and metabolic and inflammatory pathology, but the causal direction of this association is unclear. The goal of this study was to examine potential bidirectional associations between advanced epigenetic age and metabolic and inflammatory markers over time in a longitudinal cohort of 179 veterans with a high prevalence of posttraumatic stress disorder (PTSD) who were assessed over the course of two years. Analyses focused on two commonly investigated metrics of advanced DNAm age derived from the Horvath (developed across multiple tissue types) and Hannum (developed in whole blood) DNAm age algorithms. Results of cross-lagged panel models revealed that advanced Hannum DNAm age at Time 1 (T1) was associated with increased (i.e., accounting for T1 levels) metabolic syndrome (MetS) severity at Time 2 (T2; *p* = < 0.001). This association was specific to worsening lipid panels and indicators of abdominal obesity ( *p* = 0.001). In contrast, no baseline measures of inflammation or metabolic pathology were associated with changes in advanced epigenetic age over time. No associations emerged between advanced Horvath DNAm age and any of the examined biological parameters. Results suggest that advanced epigenetic age, when measured using an algorithm developed in whole blood, may be a prognostic marker of pathological metabolic processes. This carries implications for understanding pathways linking advanced epigenetic age to morbidity and mortality.

## Introduction

Recent advances demonstrate that DNA methylation (DNAm) levels at a subset of CpG loci in the genome can be used to construct “DNAm age” scores that predict chronological age with great accuracy [1–4]. In particular, the Horvath and Hannum algorithms use methylation status from 353 CpGs and 89 CpGs (Hannum DNAm age derived from the “all data” algorithm [1]), respectively, to determine DNAm informed estimates of chronological age. These estimates indicate advanced epigenetic age when DNAm age is over-estimated relative to chronological age. The Horvath algorithm is a multi-tissue predictor [2] while the Hannum metric was developed in whole blood [1]; both show approximately equivalent associations with chronological age and are highly correlated with each other [5]. However, they seem to capture different aspects of advanced epigenetic age given that metrics of over or under-estimated DNAm age relative to chronological age are only modestly correlated with each other (*r* = 0.44 to *r* = 0.56) [5–7]. Using these metrics, advanced DNAm age has been associated with increased risk for premature death [8–12], early onset of age-related disease [13,14], changes in physical and cognitive fitness [10], and cancer [12].

Several studies have also shown associations between advanced DNAm age and factors that contribute to age-related diseases, including metabolic pathology, such as obesity [15], body mass index (BMI) [16,17]; lipid levels, and inflammation [18]. For example, in a cross-sectional study, Irvin et al. [18] investigated epigenetic age via both Horvath and Hannum algorithms and found that advanced Horvath DNAm age was associated with lower interleukin 2 receptor subunit alpha, increased postprandial high-density lipoprotein (HDL), and increased postprandial total cholesterol, whereas advanced Hannum DNAm age was associated with lower C-reactive protein (CRP), lower TNF-alpha, lower fasting HDL, and increased postprandial triglycerides (TG). These results suggest that advanced epigenetic age may be associated with a more pathological response to high-fat food consumption, which could contribute to the link between advanced epigenetic age and premature onset of cardiometabolic disorders. In another recent study, Quach et al. [17] investigated associations between lifestyle factors and multiple metrics of advanced epigenetic age in blood in postmenopausal women and in a second cohort of women and men. This study found that advanced DNAm age was cross-sectionally associated with reduced poultry intake and increased BMI. In addition, a second metric of DNAm age that incorporated immune markers into the algorithm was cross-sectionally associated with dietary fish intake, moderate alcohol consumption, education, BMI, blood carotenoid levels, and CRP levels. These results suggest that health-related behaviors are associated with markers of advanced epigenetic age and that advanced epigenetic age is associated with metabolic pathology. However, the causal direction of these associations is unclear as the majority of studies have focused on cross-sectional designs, and it is not evident if advanced epigenetic age gives rise to increasing metabolic pathology and inflammation or if metabolic pathology and inflammation contribute to advanced epigenetic age, or both.

A small number of studies have investigated associations between advanced DNAm age and biological processes in longitudinal cohorts. This is important because cross-sectional associations cannot provide information concerning the correlates of *accelerated* DNAm age (i.e., the pace of epigenetic aging over time); rather, cross-sectional studies can more accurately be thought of as identifying correlates of *advanced* epigenetic age (i.e., a snapshot of cellular age at one time point). Grant et al. [16] examined a small cohort (N=43) of women and found that epigenetic age acceleration over a 16-year time period was positively associated with subsequent BMI, and nominally associated with glucose levels, however, examination in a larger cohort across a three-year time period did not replicate these findings. Longitudinal data spanning an average of 2.7 years was available in a subset (*n*=239) of the Quach et al. [17] cohort described in the preceding paragraph, and analyses in that data suggested that changes in BMI over time were associated with changes in metrics of advanced DNAm age over time (e.g., correlated change), however, baseline BMI did not predict change in methylation age acceleration over time and the longitudinal correlates of advanced baseline epigenetic age were not investigated. Given this paucity of longitudinal data and the conflicting nature of the reports from longitudinal studies, the causal direction of association between metabolic and inflammatory pathology and epigenetic age remains unclear.

The primary goal of this study was to evaluate potential bidirectional associations between advanced epigenetic age and metabolic and inflammatory markers over time. Furthermore, as we have previously shown that symptoms of posttraumatic stress disorder (PTSD) are cross-sectionally [5–7] and longitudinally [19] associated with advanced DNAm age and that PTSD is associated with metabolic [20–22] and inflammatory pathology [23], we also included PTSD (which is highly prevalent in our veteran sample) as a predictor in our models. This allowed us to differentiate effects attributable to advanced DNAm age from those associated with PTSD, and provided new information regarding PTSD-related changes in metabolic and inflammatory markers over time. We evaluated this in a longitudinal cohort of 179 military veterans in which we have previously shown that psychiatric conditions and symptoms (including posttraumatic stress disorder [PTSD] and alcohol-use disorders) are associated with an increasing pace of epigenetic age over time [19].

## RESULTS

### Cross-lagged models: Hannum DNAm age residuals

**Figure 1:**
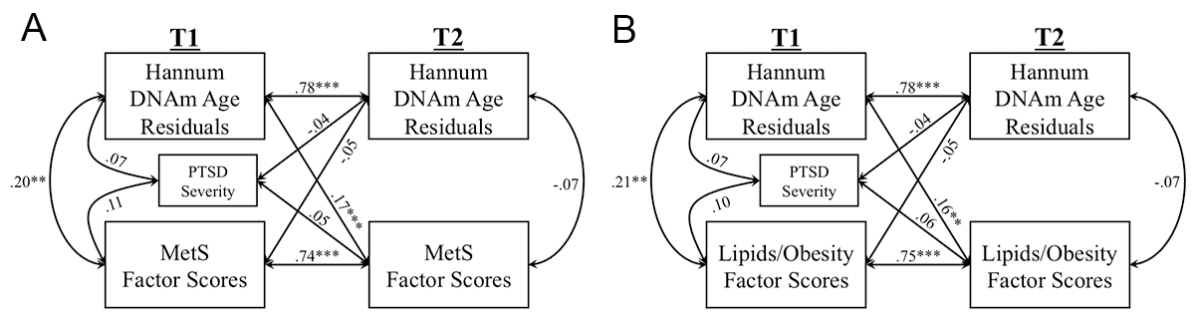
The Figure shows the results of cross-lagged models examining longitudinal associations between Hannum DNAm age residuals and metabolic syndrome (MetS) severity factor scores (A), and Lipids/Obesity factor scores (B). Measures of each marker were residualized on age and sex (applicable to A and B). (***p < 0.005, **p < 0.01, **p < 0.05).

The MetS CFA fit the data well at both T1 and T2 with all indicators loading significantly on their respective latent variables at the *p* ≤ 0.003 level (details available from corresponding author). At both time points, the Lipid/Obesity factor showed the strongest loading on the higher-order MetS factor (βs = 0.92 – 0.96) followed by the Blood Pressure factor (βs = 0.48 – 0.53), and the Blood Sugars factor (βs = 0.23 – 0.48). The cross-lagged panel analysis examining bidirectional longitudinal associations between DNAm age residuals and MetS severity revealed significant autoregressive effects between each variable and itself over time as well as a significant cross-lagged association. Advanced DNAm age at T1 predicted increases in MetS severity at T2 (standardized β = 0.17, *p* < 0.001), accounting for baseline levels of MetS (Figure 1A). T1 MetS and T1 DNAm age residuals were correlated with each other (*r* = 0.20, *p* = 0.006). Notably, the competing cross-lagged path (from T1 MetS to T2 DNAm age residuals) was not significant (standardized β = -0.05, *p* = 0.32). We examined potential confounds of the T1 DNAm age to worsening T2 MetS association and found that this association remained significant when additionally controlling for demographic factors (race, education), psychiatric factors (cigarette use, major depression, alcohol abuse/dependence), medication use (including psychotropic and metabolic-related medications), and time between assessments (see Supplementary Materials). Of note, in these follow-up analyses, major depressive diagnoses at T1 also predicted increasing MetS severity over time (standardized β = 0.28, *p* = 0.035). To further ensure no influence of additional PCs on the reported results, we also ran secondary analyses investigating the potential effects of all 20 estimated ancestry PCs (see Supplementary Materials).

There were no other cross-lagged effects between any of the other peripheral biomarkers and Hannum DNAm age residuals (see Figure S1), however associations between T1 DNAm age residuals and T2 CRP (standardized β = 0.13, *p* = 0.053) and T2 WBCs (standardized β = 0.10, *p* = 0.068) just missed the threshold for statistical significance (Figure S1 A, B). When we re-analyzed these associations in follow-up models using DNAm age residuals that did not account for estimated WBCs (i.e., only age, sex, and the first two PCs were regressed out of DNAm age estimates), we found that results for measured WBCs were unchanged, but results for CRP were nominally significant, though they missed our a priori corrected *p*-value threshold (β = 0.15, *p* = 0.031; Figure S4). A significant association between T1 PTSD symptom severity and worsening CD4/CD8 t-cell profiles (e.g., controlling for baseline CD4/CD8) emerged in that model (standardized β = -0.11, *p* = 0.013; Figure S1C).

Given that the MetS factor scores were derived from a higher-order CFA, we wondered if the association between advanced DNAm age at T1 and increasing MetS at T2 would be evident in each of the lower-order factors that comprise MetS. Therefore, we conducted additional cross-lagged models in which each of the lower-order metabolic factor scores (Lipids/Obesity, Blood Sugars, Blood Pressure) was included in the model in place of MetS, again residualized for age and sex. Results revealed associations only between T1 DNAm age residuals and increasing Lipid/Obesity factor scores at T2 (standardized β = 0.16, *p* = 0.001), controlling for the significant baseline effects of the Lipid/Obesity factor scores (standardized β = 0.75, *p* < 0.001; Figure 1B; see Supplementary materials for results pertaining to individual indicators of this latent variable). A T1 correlation between advanced DNAm age and Lipid/Obesity factor scores was also evident (*r* = 0.21, *p* = 0.003). No cross-lagged effects emerged with the Blood Pressure or Blood Sugar factor scores (Figure S2).

### Cross-lagged models: Horvath DNAm age residuals

We found no significant cross-lagged associations between Horvath DNAm age residuals and any of the peripheral biomarkers evaluated (Figure S3). There were also no significant concurrent correlations between Horvath DNAm age residuals and any of the peripheral biomarkers (Figure S3). As in the model with the Hannum DNAm age residuals, PTSD severity was associated with worsening CD4/CD8 T-cell profiles (Figure S3).

## DISCUSSION

This is the first study to examine potential bidirectional longitudinal associations (over the course of two years) between two measures of epigenetic age acceleration (Hannum and Horvath) and changes in peripheral metabolic and inflammatory markers in a well-assessed longitudinal cohort. A goal of the study was to understand the biological consequences of advanced epigenetic age, given that accelerated cellular age has previously demonstrated associations with a variety of diseases and early death [5,8,10–12]. We evaluated potential bidirectional longitudinal associations, given untested assumptions in prior cross-sectional studies that inflammatory and metabolic parameters are causative in accelerating epigenetic age. We found that advanced Hannum DNAm age was strongly associated with increased MetS-related pathology approximately two years later (Figure 1A). Follow-up investigations revealed that the observed effect on MetS was specific to increased (more pathological) scores on the Lipid/Obesity factor (defined by BMI, waist/hip ratio, HDL, and TG (Figure 1B), suggesting that advanced DNAm age may lead to, or serve as a useful prognostic indicator of, increasing obesity and pathological lipid profiles, a risk factor for many age-related diseases [20,24]. In contrast, there was no evidence for the reverse direction of association (competing cross-lagged path); neither MetS nor Lipid/Obesity factor scores at T1, nor any other biological parameters investigated, predicted changes in DNAm age residuals at T2.

Our findings are consistent with recent studies that have observed significant cross-sectional associations between DNAm age acceleration and BMI [15–17] lipid levels [18] and acute increases in triglyceride levels following a high-fat food challenge [18]. Quach et al. [17] also found that increases in BMI across a two-year period (but not initial BMI) were associated with increased epigenetic age at the follow-up two-year time point. Our results are, to our knowledge, the first to demonstrate that advanced epigenetic age at a baseline assessment is associated with increasing lipid and obesity-related parameters over time. The longitudinal nature of our study allowed us to untangle the temporal relationship between these parameters and lends support for a unidirectional association. These findings add to a broader literature demonstrating that MetS is an important clinical correlate of accelerated aging; MetS has also been associated with other markers of accelerated aging, including shorter telomere length [25] though in those studies, obesity predicted decreased telomere length over time, and this was found to be driven by insulin resistance [26]. Our results highlight MetS, and specifically Lipid/Obesity-related factors, as: 1) a critical correlate of advanced cellular aging that may be indicative of a state of biochemical stress fueling negative health outcomes [27] and 2) a potential target for therapeutic intervention to reverse cellular aging. Notably, dietary and caloric restriction has been strongly associated with lifespan extension, changes in DNAm, and is one of the most robust predictors of slowed aging [28–30]. Further, our results emphasize that advanced DNAm age at baseline predicts worsening MetS pathology over and above the effects of baseline MetS, highlighting the potential clinical utility of DNAm age estimates.

Our results also raised the possibility that advanced DNAm age may be associated with increased inflammatory responses, given that associations between advanced Hannum DNAm age and increasing CRP and WBC levels just missed the threshold for statistical significance (Figure S1 A, B). These results were essentially unchanged regardless of whether estimated WBC types were accounted for in the DNAm age residuals and highlight the need for additional research to further examine this association. An association between advanced epigenetic age and CRP would be consistent with existing research; for example, Quach et al. [17] found that advanced DNAm age was associated with greater CRP levels in a cross-sectional study, and Irvin et al. [18] found that advanced Hannum DNAm age was associated with elevated levels of the inflammatory markers CRP, IL2sRa, TNFa, and MCP1. Higher WBC and CRP levels have been previously associated with age-related chronic diseases [31]. Aging has been associated with decreased innate immune system responding (i.e., immunosenescence [32–35]), as evidenced by poorer vaccine responses and loss of acquired immunity to pathogens, and with increased chronic inflammation, as evidenced by elevated pro-inflammatory markers. “Inflamm-aging” refers to this chronic and heightened pro-inflammatory profile [36,37]. Increased epigenetic age has consistently been linked to adverse health outcomes, and additional research in larger samples is needed to further evaluate if advanced DNAm age leads to reduced integrity of the immune and inflammatory systems (and not the reverse direction).

We also found that PTSD symptom severity at T1 predicted decreasing (i.e., worsening) CD4/CD8 T-cell ratios at T2 (Figure S1C, S3C). The ratio of CD4 to CD8 T-cells has been consistently used as a marker of dysregulated immune function and immunosenescence. A low ratio of CD4 to CD8 T-cells is indicative of decreases in naïve T-cells and increases in differentiated memory T-cells, which indicate a senescent T-cell phenotype [38,39]. CD4/CD8 T-cell ratios have been associated with increased morbidity and mortality [40], and low CD4/CD8 T-cell ratios have been shown to predict mortality over a four-year time period [39]. Our findings are consistent with a cross-sectional study demonstrating significantly reduced CD4/CD8 ratios in individuals with PTSD [41], and further suggest that PTSD contributes to pathological changes in basic immune parameters over time. Trauma exposure and PTSD have been associated with increased risk for autoimmune and inflammatory diseases [41,42], and our results raise the possibility that alterations in CD4/CD8 ratio are a factor linking PTSD with these health conditions.

We observed significant longitudinal associations between advanced epigenetic age and peripheral biomarkers as measured via the Hannum algorithm; however, there were no effects of Horvath DNAm age residuals on biological parameters at T2. A primary difference between the two DNAm age algorithms is that the Hannum metric was developed in whole blood whereas the Horvath metric was designed to be a multi-tissue age predictor. The Hannum metric may be more sensitive to pathological changes in blood, potentially accounting for the variability in results across the two metrics. Other studies, including those from our group, have observed differing results across DNAm age predictors and have previously suggested that they may each be sensitive to different underlying biological processes [3,19].

Results carry implications for those seeking to identify subtle yet important shifts in an individual’s underlying biology that may be a marker for increasing metabolic pathology over time. More specifically, the findings reported here were observed while including MetS at T1 in the model, suggesting that advanced DNAm age can provide unique and additional information regarding individuals at risk for worsening MetS pathology, beyond what is evident from baseline MetS parameters. Our longitudinal results also raise the possibility that the Hannum DNAm age index will be valuable for monitoring meaningful biological outcomes and tracking responses to interventions across time. Early detection and identification of individuals with high risk could allow for earlier targeted interventions focused on metabolic health.

### Study limitations

Results from this study should be interpreted with several limitations in mind. First, the study cohort was composed primarily of white male veterans. Future work is needed to establish that these results generalize to populations with more diverse compositions of ancestry and sex. Second, other biological variables that were not interrogated here may also play an important role. For example, unmeasured third variables (e.g. physical health diagnoses) could account for the predictive effects of either DNAm age residuals or PTSD on biological changes at T2. That said, this was a young adult cohort and individuals with neurological diseases and diabetes were excluded, which attenuates this concern. It remains to be seen if DNAm age residuals at T1 play a causative or etiological role in predicting negative health outcomes at T2, or if they are simply a marker for an underlying biological process. Third, though longitudinal, our study only covered a two-year period; our results should be interpreted with this in mind, as some associations of epigenetic aging and biological markers may not be detectable across this relatively short period. However, the unreliability in DNAm age estimates would be expected to be more impactful when examining change in DNAm age estimates over a relatively short compared to long period of time. Furthermore, DNAm age for individuals in the sample increased, on average, approximately one year for every chronological year, providing greater confidence that changes in DNAm age and DNAm age residuals over time are meaningful and not a function of error. Finally, our study was limited to analysis of DNAm and biological parameters in blood; we did not investigate tissue-specific cellular aging in the brain or other organs.

## Conclusion

This study provides a longitudinal investigation of the bidirectional association between cellular age as assessed by DNAm age, and key peripheral metabolic and inflammatory biomarkers. We found that DNAm age is associated with increasing MetS two years later, and furthermore, that this association is specific to increasing markers of Lipids/Obesity (Figure 1). Importantly, the longitudinal study design allowed us to show that while advanced epigenetic age predicts increasing metabolic pathology, there were no significant contributions of metabolic (or inflammatory) pathology to accelerated epigenetic age over time. These results could inform targeted lifestyle interventions that make use of DNAm age as a way to identify individuals at risk for worsening metabolic pathology and track their responses to health-promoting interventions. Altogether, our results suggest that metabolic and inflammatory processes may be key biological mechanisms by which advanced epigenetic age is associated with age-related health outcomes and problems.

## METHODS

### Participants

Participants were previously described in Wolf et al. [19]. They were U.S. military veterans (post-9/11 conflicts) who enrolled in the Translational Research Center for TBI and Stress Disorders (TRACTS) longitudinal study at the US Department of Veterans Affairs (VA) Rehabilitation Research and Development Traumatic Brain Injury Center of Excellence at VA Boston Healthcare System. The TRACTS longitudinal study has been described in detail previously [43]. In brief, it is an ongoing research protocol evaluating traumatic stress, traumatic brain injury (TBI), health, and neural and cognitive factors among returning veterans. Exclusion criteria for the study included the following: history of seizures unrelated to head injury, severe or unstable psychological diagnosis preventing participation, acute psychotic or bipolar disorder, neurological illness, acute homicidal and/or suicidal ideation with intent to act, and cognitive disorder due to general medical condition not related to TBI. As in Wolf et al. [19], the cohort investigated in this study was based on a subset of 179 TRACTS participants with DNAm data from two time points, Time 1 (T1) and Time 2 (T2), at the time of the second data freeze when DNA was processed. The clinical and sociodemographic characteristics of the study cohort are shown in Table 1.

**Table 1:** Demographic and clinical characteristics of the longitudinal sample.

**Variable**	**T1 Mean (SD)**	**T2 Mean (SD)**	**% (n)**
**Chronological age (years)**	33.31 (9.25)	35.20 (9.19)	
**Years between T1 and T2**	1.89 (0.65)		
**Sex (male)**			88.3 (158)
**Race**			
**White**			74.9 (134)
**Black**			9.6 (17)
**Latino/a**			12.4 (22)
**Asian**			1.7 (3)
**American Indian**			0.6 (1)
**Education**			
**High school grad or less**			31.8 (57)
**Some college or completed college**			68.2 (122)
**Beyond college**			0.0 (0)
**Cigarette smoking (Yes)**			42.0 (23.5)
**Current PTSD symptom severity**	47.40 (28.42)	45.57 (30.30)	
**Measured White blood cell counts**	6.27 (1.63)	6.50 (1.78)	
**Estimated CD4/CD8**	2.50 (1.83)	2.90 (2.76)	
**CRP^a^**	0.219(0.416)	0.317 (0.549)	
**Metabolic syndrome (MetS)^b^**	-0.025(0.049)	0.000023 (0.047)	
**Blood pressure (mm Hg)^c^**			
**Systolic**	116.4 (12.4)	121.85 (12.2)	
**Diastolic**	76.55 (9.55)	79.13 (9.75)	
**Lipid/obesity^d^**			
**HDL Cholesterol (mg/dL)**	47.7 (11.1)	48.4 (12.8)	
**Waist-to-hip ratio**	0.881 (0.074)	0.890 (0.078)	
**BMI^f^**	28.0 (4.31)	28.8 (4.60)	
**Triglycerides (mg/dL)^g^**	138.0 (129.5)	138.6 (95.7)	
**Blood sugar^h^**			
**Fasting glucose (mg/dL)^i^**	85.6 (11.7)	92.4 (9.54)	
**A_1c_ (% of hemoglobin)**	5.36 (0.273)	5.42 (0.326)	

*Note*. SD = standard deviation; T1 = time point 1; T2 = time point 2; PTSD = posttraumatic stress disorder; CRP = C-reactive protein; HDL = high-density lipoprotein; BMI = body mass index. Missing observations: Current PTSD symptom severity (T2) (*n*=1), measured WBC counts (T1) (*n*=3), measured WBC counts (T2) (*n*=5), estimated CD4/CD8 (T1) (*n*=6), estimated C4/CD8 (T2) (*n*=6), CRP (T1) (*n*=5), CRP (T2) (*n*=6), MetS (T1) (*n*=1), MetS (T2) (*n*=2), systolic blood pressure (T2) (*n*=5), diastolic blood pressure (T2) (*n*=5), HDL cholesterol (T1) (*n*=7), HDL cholesterol (T2) (*n*=7), waist-to-hip ratio (T1) (*n*=6), waist-to-hip ratio (T2) (*n*=6), BMI (T2) (*n*=7), triglycerides (T1) (*n*=5), triglycerides (T2) (*n*=4), fasting glucose (T1) (*n*=4), fasting glucose (T2) (*n*=6), A_1c_ (T1) (*n*=2).^a^CRP values reported above are raw values. logCRP values were used in reported analyses. ^b^Metabolic syndrome (MetS) severity was determined using confirmatory factor analysis (CFA) of raw laboratory values and physiologic measurements. The lower order factors represented: (a) blood pressure (indicated by two diastolic and systolic readings), (b) lipids/obesity (indicated by waist-to-hip ratio, body mass index [BMI], high density lipoprotein, and triglycerides); and (c) blood sugars (indicated by fasting glucose and glycated hemoglobin A1c levels). These three factors were specified to load together (i.e., to be accounted by) a higher-order factor representing overall MetS severity. ^c^Blood pressure, ^d^lipids, and ^h^sugar are reported above as raw values. Indicators of these variables were used for the MetS CFA.

### Procedure

Participants provided written informed consent, and then completed a comprehensive interview and self-report-based psychological assessment. All diagnostic interviews were administered by doctoral-level psychology professionals. A team of psychologists reviewed each interview to determine consensus ratings of presence or absence of psychological diagnoses. For each time point (T1 and T2), blood was drawn for DNA extraction and metabolic assays. T1 and T2 assessments were conducted an average of 1.89 years apart (Table 1). The study was approved by the VA Boston Healthcare System IRB. All T1 and T2 samples were processed using the Illumina EPIC chip.

### Measures

The Clinician Administered PTSD Scale for *DSM-IV* (CAPS [44],), a well-validated diagnostic interview, was used to assess PTSD status and symptom severity. The CAPS was administered by doctoral-level psychologists. Additional information regarding the administration and rating of interviews is provided in the Supplementary Materials. In this manuscript, our analyses focused on a dimensional index of current PTSD symptom severity at T1. Additional measures that were included in supplementary analyses are described in the Supplementary Materials.

### DNA extraction, genotyping, and ancestry-based principal components analysis

Full details on genotyping protocols, techniques, and data cleaning procedures are detailed in Logue et al. [45], and are also summarized here. DNA extraction was performed using a Qiagen AutoPure instrument with Qiagen reagents. DNA concentrations were normalized using the Quant-iT^TM^ PicoGreen dsDNA fluorescent assay (Invitrogen). To determine DNA quality and quantity, TaqMan RNase P Detection assay was used (Applied Biosystems Assay, Life Technologies, Carlsbad, CA) with fluorescence detection on a 7900 Fast Real Time PCR Instrument (Applied Biosystems, Life Technologies, Carlsbad, CA). DNA was then whole-genome amplified, fragmented, precipitated, resuspended, and was then hybridized on Illumina HumanOmni2.5-8 beadchips for 20 hours at 48°C according to manufacturer’s instructions (Illumina, San Diego, CA), followed by a single-base extension and multi-layered staining process. Beadchips were imaged using the Illumina iScan System, and results were processed with the Illumina GenomeStudio v2011.1 software and the Genotyping v1.9.4 module. Genotypes were then used to develop principal components (PCs) to model ancestry: PCs were determined using 100,000 randomly chosen common (minor allele frequency >5%) single nucleotide polymorphisms (SNPs) in PLINK version 1.9 [46]; PCs were used as ancestry covariates in subsequent analyses.

### Methylation

DNA was hybridized to the Infinium MethylationEPIC BeadChip per manufacturer’s instructions. T1 and T2 samples were run together on the same chip, balancing the presence of cases and controls across chips and chip positions in order to reduce systematic bias. We utilized the processing pipeline established by the Psychiatric Genetics Consortium (PGC) PTSD Epigenetics workgroup [47] for the Illumina HumanMethylation450 BeadChip updated to apply to the EPIC chip. We used GenomeStudio to derive individual-level background-corrected probe data and idat files, and cleaned DNAm data using the CpGassoc package [48] and the ChAMP package [49] in R (R Development Core Team, 2008). Individual methylation values that did not meet a detection p < 0.001 were set to missing, and probes with >10% missing values were dropped. One chip had 7 out of 8 failed samples based on a criteria of >5% missing values; data for this chip were discarded and samples were rerun on a new chip. Subsequently no samples had >5% missing data, and all were retained for analysis. No samples had intensity <50% of the experiment-wide mean or with intensity <2,000 arbitrary units (AU). Cross hybridizing probes [50] and “underperforming” EPIC probes according to Illumina Product Quality Notification PQN0223 04/19/2017 were also excluded. R v. 3.1.0 was used for data cleaning.

### Epigenetic age calculation

For Hannum DNAm age estimates, data were normalized using the beta mixture quantile dilation (BMIQ) method in the wateRmelon R package [51] as previously described [5,7,19] and batch correction was performed using an empirical Bayes method implemented in COMBAT [52]. Horvath DNAm age estimates were determined following an R script based on 335 probes assessed on the EPIC chip that passed quality control (QC). We have previously shown that DNAm age estimate correlations with chronological age are similar across the EPIC and 450K chips [7,53]. The association between Hannum DNAm age estimates derived from the EPIC chip in this cohort and chronological age was r = 0.88 (*p* < 0.001) and r = 0.85 (*p* < 0.001) at T1 and T2, respectively, and for the Horvath algorithm, the association was r = 0.90 (*p* < 0.001) and r = 0.91 (*p* < 0.001) at T1 and T2, respectively [7]. Hannum and Horvath DNAm age estimates were correlated with each other at both time points (T1, *r* = 0.88, *p* < 0.001; T2, *r* = 0.87, *p* < 0.001), as were Hannum and Horvath DNAm age residuals (*r* = 0.44, *p* < 0.001, for both T1 and T2) [19].

### MetS (lipid/obesity, blood pressure, sugar levels)

Height, weight, and waist-to-hip ratio were measured along with two standing and two seated blood pressure readings (taken at 1-minute intervals). Blood samples were obtained, processed immediately upon collection, and shipped the same day to a commercial laboratory (Quest Diagnostics, Cambridge, MA). This laboratory assessed HDL cholesterol, triglycerides, and glucose (fasting glucose and glycated hemoglobin A1c levels). These metabolic measures were included in an overall index of Metabolic Syndrome (MetS) severity using confirmatory factor analysis (CFA) as described in the analysis section below. Total white blood cell counts were also measured via complete blood chemistry.

### Estimated white blood cell count and CD4/CD8 ratio

Specific white blood cell (WBC) type proportions at T1 and T2 were not available from the Quest metrics and instead were estimated based on the methylation data. In brief, CD4 and CD8 T-cells, natural killer cells, b-cells, and monocytes were estimated based on the methylation data using the R minifi package [54] according to procedures described by Houseman et al. [55], Jaffe and Irizarry [56], and Fortin et al. [57]. CD4 and CD8 T-cell estimates were used to calculate a ratio of CD4 to CD8 T-cells, which has previously been shown to be an index of immunosenescence (see also: Holbrook et al. [58]).

### C-reactive protein (CRP) serum levels

Serum CRP was assessed in blood samples as previously described in Miller et al. [23]. Serum CRP levels were measured in a commercial laboratory (Quest Diagnostics, Cambridge, MA) using a nephelometric assay with CRP monoclonal antibodies (analytical sensitivity = 0.10 mg/dL). Laboratory assay procedures were standardized to CRP reference preparations (International Federation of Clinical Chemistry and Laboratory Medicine/Bureau Communautaire de Reference/College of American Pathologists). The sample mean at T1 was 0.19 mg/dl (SD=1.13; range: 0.09-0.69 mg/dl), and at T2 was 0.29 mg/dl (SD=0.37; range: 0.09-2.34 mg/dl). Data were log-transformed for analysis (referred to as “CRP log” due to the distribution of raw CRP values being positively-skewed as in Miller et al. [23]. Two outliers were removed (one from each time point) as their CRP estimates were 12 SDs above the mean.

### Data analysis

An overall index of MetS severity was calculated using CFA of raw laboratory values and physiologic measurements as previously described in Wolf et al. [20]. CFA is ideal for measuring the common metabolic factors that underlie the covariation of various biological assays because it models their relationship to a shared latent (or common) variable. Factor scores on the latent variable can then be generated to reflect the severity of the metabolic pathology for each subject. This approach avoids concerns about arbitrary diagnostic thresholds for metabolic disease and instead models the severity and comorbidity of the metabolic components dimensionally. As in Wolf et al. [20], we developed a higher-order CFA. The lower order factors represented: (a) Blood Pressure (indicated by two seated diastolic and systolic readings); (b) Lipid/Obesity (indicated by waist-to-hip ratio, body mass index (BMI), high density lipoprotein, and triglycerides); and (c) Blood Sugars (indicated by fasting glucose and glycated hemoglobin A1c levels). The above factors were specified to load together (i.e., to be accounted by) on a higher-order factor representing overall MetS severity. The model was tested separately in the T1 and T2 data and factor scores on all latent variables were saved for subsequent analyses. Of note, insulin was not available for the majority (94%) of the subjects at T1, as this test was added later to the protocol, and thus insulin levels were not included in the MetS CFA.

**Figure 2:**
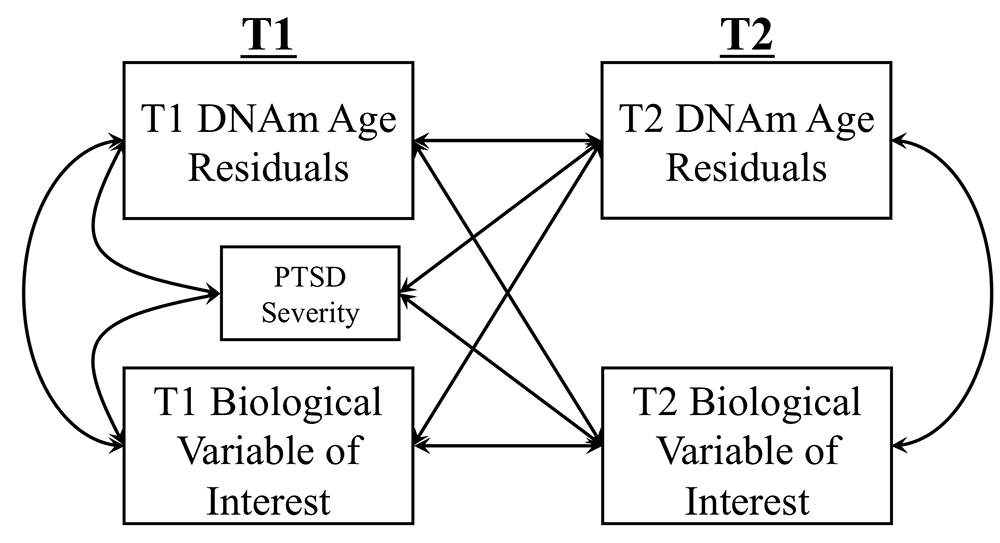
The Figure shows the cross-lagged model used to examine longitudinal associations between DNAm age residuals (Hannum or Horvath) and biological variables of interest (MetS, lab-based WBC measurement, CRP levels, CD4/CD8 T-cell ratio). Measures of each biological marker were residualized on age and sex for all analyses. DNAm age residuals at each time point were generated by regressing raw DNAm age estimates on age, sex, estimated WBCs (CD4-T, CD8-T, NK, b cells, monocytes) from the respective time point, and the top two ancestry PCs and saving the unstandardized residuals from this equation. For analyses predicting estimated CD4/CD8 ratios, DNAm age residuals were calculated by regressing raw DNAm age estimates on age, sex, and the top two ancestry PCs (but not on estimated WBCs as these were the focus of this analysis).

We conducted cross-lagged panel models (a form of path analysis) to simultaneously evaluate bidirectional longitudinal associations between advanced epigenetic age and each peripheral biomarker of interest (Figure 2). In this analysis, each variable measured at T2 is regressed on the same variable at T1 (i.e., the auto-regressive effect) and on the competing variable at T1 (i.e., the cross-lagged effect). For example, in the model examining MetS, T2 advanced epigenetic age (as defined by DNAm age residuals) was regressed on T1 DNAm age residuals and on T1 MetS factor scores while T2 MetS was simultaneously regressed on T1 MetS and T1 DNAm age residuals. The association between T1 PTSD severity and both T2 variables was also modeled. The concurrent correlations among the predictors at T1 and the residual correlation among dependent variables at T2 were also evaluated. A significant cross-lagged path would indicate, for example, that T1 Mets predicts changes in T2 advanced epigenetic age, controlling for T1 advanced epigenetic age. DNAm age residuals at each time point were generated by regressing raw DNAm age estimates on age, sex, estimated WBCs (CD4-T, CD8-T, NK, b cells, monocytes) from the respective time point, and the top two ancestry PCs and saving the unstandardized residuals from this equation. For analyses predicting estimated CD4/CD8 ratios, DNAm age residuals were calculated by regressing raw DNAm age estimates on age, sex, and the top two ancestry PCs (but not on estimated WBCs as these were the focus of this analysis). In a similar set of analyses, we also investigated measured WBCs and CRP phenotypes using Horvath and Hannum DNAm age residuals that did not take into account the estimated WBCs from DNAm age (regressing raw Horvath and Hannum DNAm estimates on age, sex, and the top two ancestry PCs, but not on estimated WBCs).

For the sake of simplicity and consistency with the DNAm age residuals, each peripheral biomarker was also first regressed on age and sex and the residuals from this equation saved for use in the path models. This approach controls for variance in these demographic covariates by removing them from both the T1 and T2 variables.

This cross-lagged panel approach was followed, in separate analyses, for each peripheral biomarker of interest (MetS, lab-based WBC totals, CRP, and estimated CD4/CD8 ratios) and for both the Horvath and Hannum algorithms. For analyses with significant cross-lagged associations, we conducted follow-up regressions which included a series of potential confounding variables to determine if they accounted for the significant cross-lagged associations, including demographic variables (education and self-reported racial/ethnic minority), psychological variables (cigarette use, current major depressive diagnosis, current alcohol abuse or dependence diagnosis), medication variables (current use of anti-hypertensives, cholesterol lowering medication, diabetes medication, antidepressants, anti-epileptics, sedatives/hypnotics, and pain medications; see Supplementary Materials), and time between assessments.

As each analysis was executed twice (once for Horvath and once for Hannum-based indices), we took into account the correlation between the two advanced epigenetic age metrics by adjusting for 1.8 tests and set the *p*-value threshold for statistical significance for individual parameters of interest (e.g., the association between DNAm age residuals and the T2 biological variable controlling for the same biological variable at T1) in a model at *p* = 0.028. This *p*-value correction was derived from a permutation testing procedure as described in Miller et al. [23] and Wolf et al. [6]; based on the *r* = 0.49 Horvarth/Hannum DNAm age residual association in the larger cross-sectional dataset, the adjusted *p*-value was found to represent 1.8 tests. There was no multiple testing correction across analyses for different biological variables of interest as these analyses investigate distinct hypotheses across different families of tests. All analyses were conducted with Mplus 8.0 statistical modeling software [59]. As all models were just identified (i.e., fully saturated such that *df* = 0), model fit will always be perfect and thus is not reported here.

## SUPPLEMENTARY MATERIAL

Supplementary Methods

Supplementary Figures
