# Supplementary material for: Investigation of bidirectional longitudinal associations between advanced epigenetic age and peripheral biomarkers of inflammation and metabolic syndrome
Source: Aging (Albany NY). 2019 Jun 7;11(11):3487–504. doi: 10.18632/aging.101992 (PMC6594822; doi:10.18632/aging.101992)
Supplement: Supplementary Methods [file aging-11-101992-s001.pdf]

SUPPLEMENTARY MATERIAL

Supplementary Methods

Supplementary Measures

The CAPS was administered by doctoral-level psychologists. The CAPS assesses the frequency and intensity of 17 symptoms of PTSD, based on DSM-IV criteria. The DSM-IV algorithm (e.g., requiring endorsement of at least 1 reexperiencing symptom, 3 avoidance and numbing symptoms, and 2 hyperarousal symptoms) was used to determine PTSD diagnoses; symptoms were considered present if item frequency ≥ 1 and intensity ≥ 2. The Structured Clinical Interview for DSM-IV Disorders [1] was administered to assess for other psychiatric diagnoses, including major depression and alcohol abuse/dependence. All diagnoses were reviewed by an expert team that included at least two psychologists to arrive at a consensus diagnosis. A dichotomous index of current cigarette use was obtained from the Fagerström Test for Nicotine Dependence (FTND) [2]. Self-reported level of education was categorized into the following groups for analysis: “high school graduation or less,” “some college or completed college,” or “beyond college” for use as covariates.

Supplementary Results

Given the significant association between advanced Hannum epigenetic age at T1 and increasing metabolic syndrome severity factor scores at T2, we conducted several additional analyses to examine potential confounds of this association. We retained the same model as that depicted in Figure 1 and added in the following additional covariates of T2 metabolic syndrome severity factor scores in four analyses: potential demographic confounds (self-reported racial/ethnic minority and education), psychiatric conditions (cigarette use, major depression, alcohol abuse/dependence), medication use (anti-hypertensives, cholesterol-lowering medication, diabetes medication, antidepressants, sedatives/hypnotics, anti-epileptics, and pain-related medications), and time between assessments.

Only one significant association between any of these variables and T2 metabolic syndrome severity factor scores emerged: major depressive disorder diagnoses at T1 were associated with worsening metabolic syndrome severity scores at T2, (standardized $\beta = 0.28$, $p = 0.035$), though Hannum DNAm age residuals were also still significantly associated with worsening metabolic syndrome scores in the same model (standardized $\beta = 0.18$, $p < 0.001$). In all other covariate models, the covariates were not associated with T2 MetS while Hannum DNAm age residuals remained significant ($p \leq 0.001$).

Across all analyses we have included the top two principal components (PCs) to control for ancestry within this cohort of white, non-Hispanic subjects. To further ensure no influence of additional PCs on the reported results, we first investigated all 20 PCs predicting Hannum DNAm age at T1 and T2, controlling for age, sex, and WBCs, and found that none of the PCs were significantly associated with Hannum DNAm age at either time point. We also retained the same model as that depicted in Figure 1A using a new Hannum DNAm age residual which was residualized for all 20 PCs, age, sex, and WBCs for both time points. Using this new Hannum DNAm age residual variable, we re-analyzed the cross-lagged model and found no change in the reported results; Hannum DNAm age at T1 (residualized for all 20 PCs, age, sex, and WBCs) significantly predicted MetS at T2 (controlling for MetS at T1; standardized $\beta = 0.15$, $p = 0.001$).

To further investigate individual indicators of the lower-order Lipid/Obesity latent variable, we residualized each of the individual indicators (BMI, waist-to-hip ratio [WHR], HDL cholesterol, and triglycerides) at each time point on age and sex and re-analyzed the cross-lagged models (retaining the same model as that depicted in Figure 1A). Hannum DNAm age residuals at Time 1 predicted increasing BMI ($\beta = 0.14$, $p = 0.003$), WHR ($\beta = 0.19$, $p < 0.001$), and triglycerides ($\beta = 0.13$, $p = 0.026$), and there was a trending, though non-significant, effect for HDL ($\beta = -0.10$, $p = 0.073$) in the expected direction. Thus, results suggested that multiple obesity-related metabolic components showed worsening profiles over time as a function of advanced DNAm age at time 1.

Supplementary References

1. First MB, Gibbon M, Spitzer RL, Benjamin LS. (1997) User's guide for the structured clinical interview for DSM-IV axis II personality disorders: SCID-II. Washington, D.C.: American Psychiatric Press, Inc.

2. Heatherton TF, Kozlowski LT, Frecker RC, Fagerström KO. The Fagerström Test for Nicotine Dependence: a revision of the Fagerström Tolerance Questionnaire. Br. J. Addict. 1991; 86: 119- 27. PMID:1932883

3. Weathers FW, Ruscio AM, Keane TM. Psychometric

properties of nine scoring rules for the Clinician-Administered Posttraumatic Stress Disorder Scale. Psychological Assessment. 1999; 11: 24.
http://dx.doi.org/10.1037/1040-3590.11.2.124
