# Supplementary material for: Investigation of bidirectional longitudinal associations between advanced epigenetic age and peripheral biomarkers of inflammation and metabolic syndrome
Source: Aging (Albany NY). 2019 Jun 7;11(11):3487–504. doi: 10.18632/aging.101992 (PMC6594822; doi:10.18632/aging.101992)
Supplement: Supplementary Figures [file aging-11-101992-s002.pdf]

Supplementary Figures

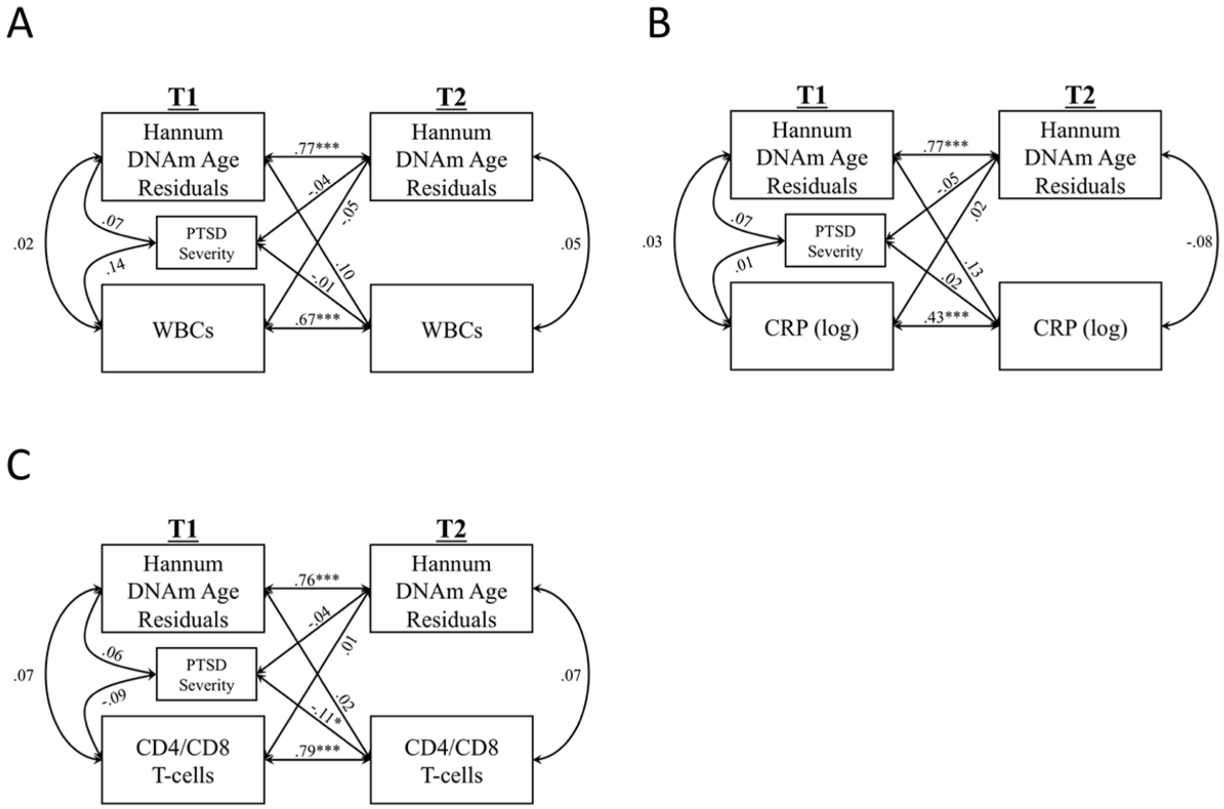

Figure S1. The Figure shows the results of cross-lagged model models examining longitudinal associations between Hannum DNAm age residuals and measured white blood cells (**A**), C-reactive protein (**B**), and CD4/CD8 t- cell ratios (**C**). Results for metabolic syndrome severity factor scores can be found in Figure 1. Measures of metabolic and inflammatory markers were residualized on age and sex (applicable to all figures). ***$p < 0.005$, **$p < 0.01$, **$p < 0.05$.

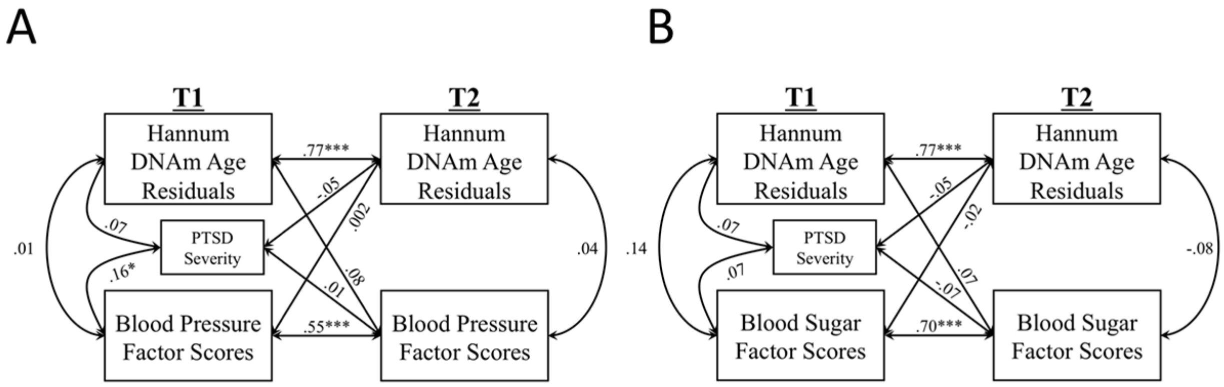

Figure S2. The Figure shows the results of cross-lagged models examining longitudinal associations between Hannum DNAm age residuals and Blood Pressure (**A**) and Blood Sugar (**B**) factor scores. Results for the Lipids/Obesity factor scores can be found in Figure 1. ***$p < 0.005$, **$p < 0.01$, **$p < 0.05$.

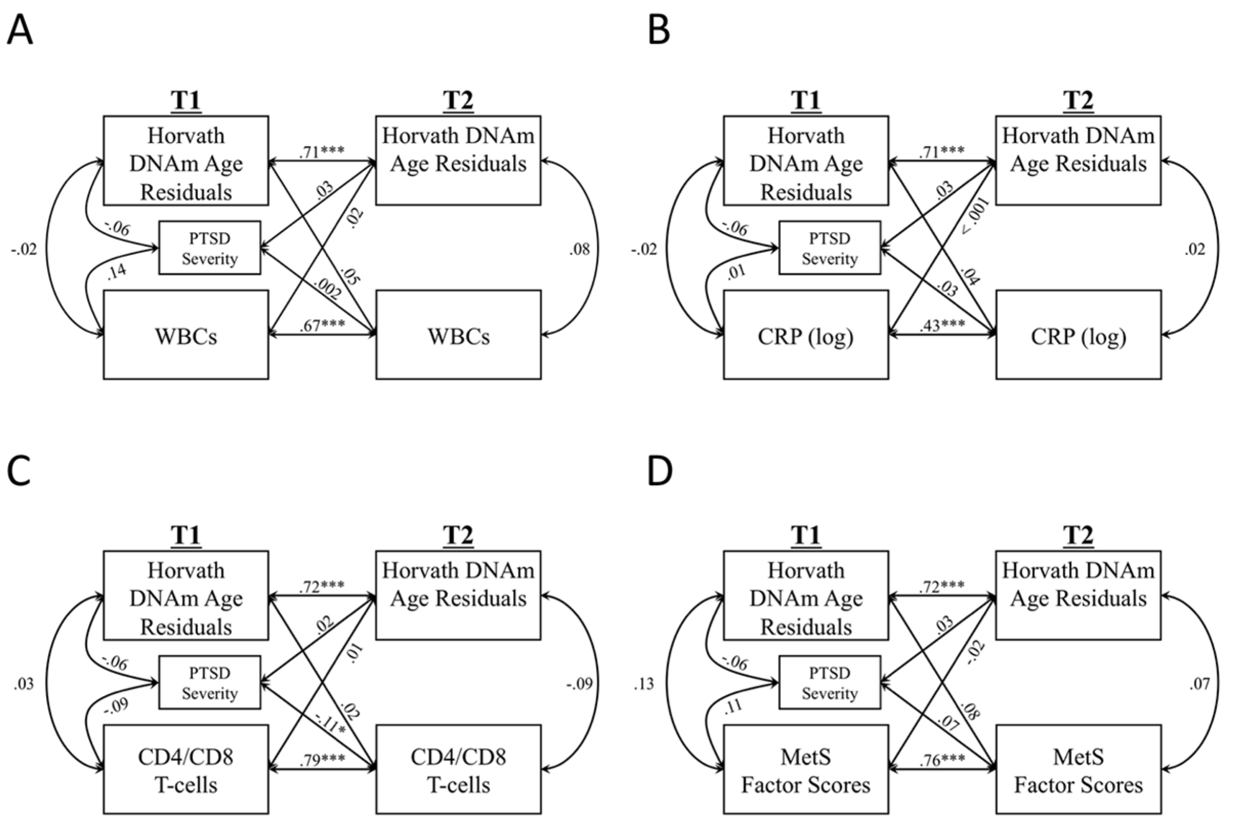

Figure S3. The Figure shows the results of cross-lagged model models examining longitudinal associations between Hannum DNAm age residuals and measured white blood cells (**A**), C-reactive protein (**B**), CD4/CD8 t- cell ratios (**C**), and metabolic syndrome severity factor scores (**D**). ***$p < 0.005$, **$p < 0.01$, **$p < 0.05$.

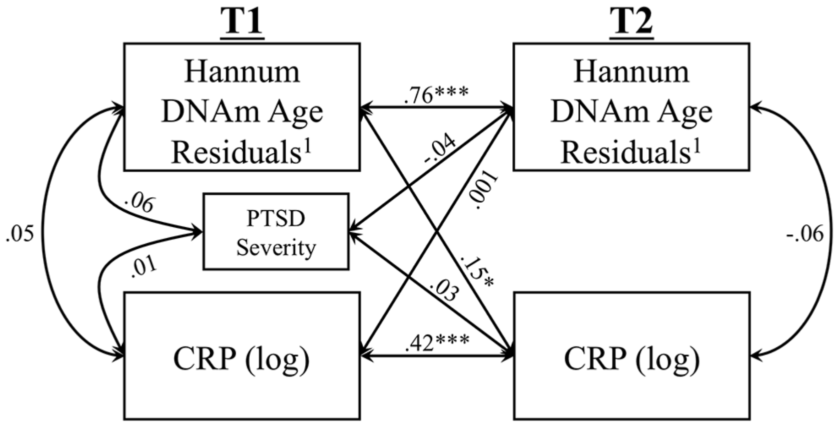

Figure S4. The Figure shows the results of cross-lagged model models examining longitudinal associations between Hannum DNAm age residuals and C-reactive protein. [1]Hannum DNAm age residuals for each time point were generated by regressing raw DNAm age estimates on age, sex, and the top two ancestry PCs and saving the unstandardized residuals from this equation (estimated WBCs were not included). ***p <0.005, **$p < 0.01$, **$p < 0.05$.
